# Associations of Academic Study- and Non-Study-Related Sedentary Behaviors with Incident Obesity in Children and Adolescents

**DOI:** 10.3390/nu17101633

**Published:** 2025-05-09

**Authors:** Tingyu Lu, Meng Li, Ruihang Zhang, Ruiqiang Li, Shaojun Shen, Qiuxia Chen, Rong Liu, Jiao Wang, Yabin Qu, Lin Xu

**Affiliations:** 1 School of Public Health, Sun Yat-sen University, Guangzhou 510080, China; luty5@mail2.sysu.edu.cn (T.L.);; 2 Guangdong Provincial Center for Disease Control and Prevention, 160 Qunxian Road, Guangzhou 511430, China

**Keywords:** sedentary behavior, overweight, obesity, children and adolescents

## Abstract

**Objective**: To assess the associations between academic study- and non-study-related sedentary behaviors and the risk of overweight/obesity in children and adolescents, as well as their joint association with sugar-sweetened beverage (SSB) consumption. **Methods**: Sedentary behaviors and SSB consumption were assessed using self-reported questionnaires. Overweight/obesity were defined by age- and sex-specific body mass index cut-off values according to the criteria of “Screening for overweight and obesity among school-age children and adolescents” in China. Poisson regression with robust error variance was used to assess the associations of sedentary behaviors and/or SSB consumption with the risk of overweight/obesity, yielding relative risks (RRs) and 95% confidence intervals (CIs). The Shapley additive explanations (SHAP) method was used to rank the contribution of five specific sedentary behaviors to obesity risk. **Results**: Among 47,148 participants with a 3-year follow-up, longer durations of screen-related, academic study-related, and total sedentary time were each associated with a higher risk of overweight/obesity (adjusted RR (95% CI) per hour increment: 1.01 (1.00–1.02), 1.03 (1.01–1.06), and 1.02 (1.01–1.03)). After mutual adjustment, the associations of engaging in homework, attending tutorial classes, and using mobile electronic devices remained significantly associated with higher overweight/obesity risk. The SHAP summary plot shows that using mobile electronic devices, attending tutorial classes, and doing homework were the three most important sedentary obesogenic contributors. A significant interaction of age with sedentary time was found (*p* for interaction < 0.05). No significant interaction was found between SSB consumption and sedentary time. **Conclusions**: Excessive sedentary behaviors were associated with a higher risk of overweight/obesity, particularly due to mobile electronic device use, attending tutorial classes, and doing homework.

## 1. Introduction

With the rapid development of the economy, childhood and adolescent obesity has emerged as a significant public health issue. According to the World Health Organization (WHO), the global prevalence of overweight/obesity among children and adolescents aged 5–19 years increased dramatically from 8% in 1990 to over 20% in 2022 [1]. In China, between 2015 and 2019, the prevalence of overweight and obesity in children and adolescents aged 6–17 years was reported to be 11.1% and 7.9%, respectively [2]. Obesity during childhood and adolescence is not only detrimental to immediate health, including adverse physical [3], psychological [4], and social consequences [5] but also increases the risk of various chronic diseases in adulthood such as cardiovascular disease [6] and type 2 diabetes [7]. Hence, identifying risk factors and implementing preventive strategies of obesity at an early age is of great importance.

Previous studies have identified multiple risk factors for childhood and adolescent obesity, including sugar-sweetened beverage (SSB) consumption [8] and excessive sedentary behavior [9]. Since sedentary behavior is often accompanied by high-calorie foods (such as snacks) [10], reduced energy expenditure [10], and solidified behavioral patterns [11], it was considered a key factor leading to obesity in children and adolescents. A recent systematic review of 17 studies involving school-age Chinese children showed the adverse influence of sedentary behavior on obesity, although it noted limitations such as cross-sectional design (12/17), and small sample sizes in cohort studies (up to 3717 children) [9]. To note, the existing studies exploring the associations between sedentary behaviors and obesity predominantly focus on screen time (12/17), with limited attention given to academic study-related sedentary behavior (5/17). Given the increasing academic pressure and widespread use of electronic devices among children and adolescents, particularly in China [12], separately examining academic study-related and screen-related sedentary behaviors is crucial to identify the most significant obesogenic behaviors and develop targeted interventions. Moreover, although specific sedentary behaviors were highly correlated with each other [13], no studies have assessed the independent associations of specific sedentary behaviors with overweight/obesity risk by mutually adjusting for other sedentary behaviors. In addition, to date, no studies have ranked the importance of these behaviors to determine the primary contributors to obesity, highlighting the need for studies exploring in depth to develop targeted interventions.

Furthermore, increasing evidence supports the co-occurrence of long sedentary time and unhealthy dietary habits, specifically the SSB consumption [14,15]. Both behaviors are classified as energy balance-related behaviors (EBRBs) that can influence energy balance and contribute to obesity [16]. The co-occurrence of SSB consumption and sedentary behavior may exacerbate their individual effects on obesity. However, no studies to date have reported the joint association of sedentary behavior and SSB consumption with the risk of overweight/obesity, warranting further investigation.

Hence, based on a representative surveillance project in Guangdong province, we conducted a 3-year longitudinal analysis to comprehensively examine the associations of academic study-related and screen-related sedentary behaviors with overweight/obesity incidence, and the independent influences of specific sedentary behaviors by mutual adjustment. Additionally, we aimed to rank the importance and identify the primary obesogenic sedentary behaviors. Moreover, we also explored the joint association between sedentary time and SSB consumption in participants aged 6–20 years.

## 2. Materials and Methods

### 2.1. Study Sample

This study was based on the “Surveillance for common disease and health risk factors among students” in Guangdong province from 2019 to 2022, which used a stratified multistage sampling method to select students in order to ensure representativeness across the province’s diverse urban and rural landscapes. Briefly, all 21 prefecture-level cities within the province were included, with one urban district and one county being selected through random sampling in each city. Within the selected urban district, a total of eight schools were chosen, comprising two each of primary, middle, and regular high schools, alongside one vocational high school and one university. Meanwhile, each rural county included five randomly selected schools: two primary schools, two middle schools, and one regular high school. We included all educational stages from the first grade in primary schools to the third year at universities, with at least two classes per grade being selected to ensure a broad capture of the target population. Details of the surveillance design are shown in Figure S1. Demographic characteristics and lifestyle factors were collected using a self-reported questionnaire, which has been tested and shown good validity and reliability [17]. Anthropometric parameters and blood pressure were measured by trained nurses and doctors using standardized procedure. After data collection, all personal identifiers were removed to maintain participant anonymity, resulting in a de-identified dataset. The study was granted exemption by the Medical Research Ethics Review Committee of the Guangdong Provincial Center for Disease Control and Prevention. Due to the small sample size of individuals younger than 6 years or older than 20 years, only students aged 6–20 years were included in this study.

### 2.2. Exposure Variables

The exposure variables were sedentary behaviors and SSB consumption, assessed by a self-reported questionnaire. For sedentary behaviors, students reported the time spent on specific activities, including doing homework, attending after-school tutorial classes (such as Chinese, math, and English), watching TV, using a computer, and using mobile electronic devices. Students were asked “How long did you spend on doing homework (per day)/attending tutorial classes (per week) after school in the past week (x)?” with response options of <1 (0 ≤ x < 1), 1+ (1 ≤ x < 2), 2+ (2 ≤ x < 3), and 3+ (x ≥ 3) h. The durations of doing homework and attending tutorial classes were combined to calculate academic study-related sedentary time (h/day). Additionally, students reported their daily time spent watching TV and using a computer in the past week (x), with response options of 0, <1 (0 < x < 1), 1+ (1 ≤ x < 2), 2+ (2 ≤ x < 3), 3+ (3 ≤ x < 4), and 4+ (x ≥ 4) h/day. Due to the small sample sizes in the groups of 3 ≤ x < 4, and x ≥ 4 h/day, these were combined into 0, <1 (0 < x < 1), 1+ (1 ≤ x < 2), and 2+ (x ≥ 2) h/day for analytic purposes. Participants also reported the average time spent using mobile electronic devices per day in the past week as a continuous variable. The durations of watching TV, using a computer, and using mobile electronic devices were summed to determine screen-related sedentary time (h/day). Total sedentary time was calculated as the sum of academic study-related and screen-related sedentary time (h/day). SSB consumption was assessed by asking participants “How often do you drink SSB such as cola, sweetened tea, fruit drinks and other sugary beverages?”, with response options of “Never drink”, “<1 time/day”, and “1+ time/day”.

### 2.3. Outcomes

The primary outcome was overweight/obesity incidence, and the secondary outcome was body mass index (BMI). Participants’ height (measured to the nearest 0.1 cm) and weight (measured to the nearest 0.1 kg) were measured by trained nurses and doctors using standardized equipment and procedures, with shoes removed. BMI was calculated as weight in kilograms divided by height in meters squared (kg/m^2^). Overweight and obesity were defined using the age- and sex-specific BMI cut-off values according to the criteria of “Screening for overweight and obesity among school-age children and adolescents” in China (Table S1) [18].

### 2.4. Potential Confounders

As sedentary behaviors, SSB consumption and overweight/obesity can be influenced by sex, age, social-economic position (ethnicity, economic level, and living in an urban/rural area) [19,20], personal lifestyle factors (smoking status, alcohol use, physical activity, and sleep duration) [20,21,22,23], and chronic disease history (hypertension, diabetes, and cardiovascular disease (CVD)), these variables were considered potential confounders and adjusted in the analyses. Additionally, consumption of various food groups (i.e., dessert, fried food, fruit, and vegetable) were adjusted for in the analyses of SSB consumption.

### 2.5. Statistical Analysis

One-way analysis of variance (ANOVA) and chi-square test were used to compare baseline continuous and categorical variables by follow-up overweight/obesity status. Specific sedentary behaviors and SSB consumption were analyzed categorically with the lowest group serving as the reference, while screen-related, academic study-related, and total sedentary time were analyzed continuously, based on per hour increments. Regarding the joint association, first, total sedentary time was categorized as a binary variable, i.e., short (below the median value of the study sample) or long (at or above the median level) sedentary time. Second, participants were categorized into six groups based on the combination of SSB consumption and total sedentary time: (1) never drink and short sedentary time; (2) never drink and long sedentary time; (3) drinking <1 time/day and short sedentary time; (4) drinking <1 time/day and long sedentary time; (5) drinking 1+ time/day and short sedentary time; and (6) drinking 1+ time/day and long sedentary time.

Poisson regression with robust error variance was used to assess the associations of sedentary behaviors and SSB consumption with overweight/obesity incidence, yielding relative risks (RRs) and 95% confidence intervals (CIs) [24]. Linear regression was used to examine the associations of baseline sedentary behaviors and SSB consumption with follow-up BMI, yielding βs and 95% CIs. We examined the interactions between total sedentary time and SSB consumption on overweight/obesity incidence or BMI, and conducted joint association analyses. We used three models to investigate the robustness of the associations, including a crude model, model 1 (adjusted for sex, baseline age, ethnicity, urban/rural area, economic level, smoking status, alcohol use, moderate-to-vigorous physical activity (MVPA) frequency, sleep duration, chronic disease history, and food groups intake (for SSB consumption only)) and mutually adjusted model 2 (i.e., mutually adjusted for SSB consumption and total sedentary time in the analyses of sedentary behaviors and SSB consumption, and added mutually adjustments in the analyses of specific sedentary behaviors). Correlation coefficients were calculated to examine whether there were strong correlations among variables included in the mutually adjusted model.

Additionally, we used extreme Gradient Boosting (XGBoost) to assess the importance of five specific sedentary behaviors contributing to overweight/obesity incidence. XGBoost, a supervised machine learning and data mining tool, which involves a meta-algorithm, to construct a strong ensemble learner from weak learners, such as regression trees. The XGBoost algorithm indicates the contributions of each of the predictors [25,26]. We further applied the Shapley additive explanations (SHAP) method to elucidate the significance of individual variables within the context of model interpretation [27]. Shapley values were determined to quantify the contribution of each variable to the overall performance of the model [28].

Several sensitivity analyses were conducted to examine the robustness of the results. First, we examined interactions of sedentary time or SSB consumption with some potential moderators (e.g., sex and age) on overweight/obesity incidence or BMI [29] and conducted subgroup analyses stratified by these moderators. Second, we separately analyzed the associations of sedentary behaviors and SSB consumption with overweight incidence or obesity incidence. Third, we used the criteria from International Obesity Task Force (IOTF) to redefine overweight/obesity and repeated the main analyses. Additionally, we used Cohen’s effect sizes to compare the baseline characteristics by participants included and excluded in our analysis. Stata/MP 17.0 and R 4.2.3 were used for data analyses. All tests were two-sided, and a *p* < 0.05 was considered statistically significant.

## 3. Results

### 3.1. Baseline Characteristics

Of 117,862 participants aged 6–20 years who had both baseline and follow-up BMI data, 63,139 participants completed baseline questionnaire (2019–2021). After excluding those with missing or unreliable information on baseline total sedentary time (*N* = 5254), and those with overweight/obesity at baseline (*N* = 10,737), 47,148 participants were included in the main analysis of total sedentary time on overweight/obesity incidence (Figure S1). The mean baseline age was 12.39 (standard deviation (SD) = 2.86) years. During an average follow-up of 1.62 (SD = 0.75) years, 2951 participants developed overweight/obesity. Table 1 shows that participants who had incident overweight/obesity were younger, had higher proportions of men, and urban residence, higher MVPA frequency, longer sleep duration, and higher BMI (all *p* < 0.05). No significant differences in ethnicity, economic level, smoking status, alcohol use, prevalence of diabetes, hypertension, or CVD were found.

### 3.2. Associations of Sedentary Behaviors and SSB Consumption with Overweight/Obesity Incidence and BMI

Table 2 shows that after adjusting for sex, baseline age, ethnicity, urban/rural area, economic level, smoking status, alcohol use, MVPA frequency, sleep duration, and chronic disease history, longer screen-related, academic study-related, and total sedentary times were associated with a higher risk of overweight/obesity (RR (95% CI) per hour increment: 1.01 (1.00–1.03), 1.03 (1.01–1.06) and 1.02 (1.01–1.03), respectively). For specific academic study-related sedentary behaviors, compared with doing homework of <1 h/day, those who spent 1+ or 3+ h/day on homework had a higher risk of overweight/obesity (RR (95% CI): 1.10 (1.01–1.21) and 1.14 (1.02–1.28)). Compared with attending tutorial classes for <1 h/week, those who spent 3+ h/week had a 17% higher risk of overweight/obesity (RR (95% CI): 1.17 (1.05–1.31)). Regarding screen-related sedentary behaviors, compared with those who never used mobile electronic devices, those who spent 2+ h/day using mobile electric devices had a 20% higher risk of overweight/obesity (RR (95% CI): 1.20 (1.08–1.33)). With additional adjustment for food groups intake, compared with no SSB consumption, more frequent SSB consumption was non-significantly associated with a higher incidence of overweight/obesity.

With similar adjustments, longer screen-related, academic study-related and total sedentary time were associated with higher BMI, with β (95% CI) per hour increment being 0.03 (0.02, 0.04), 0.12 (0.10, 0.14) and 0.04 (0.03, 0.05) kg/m^2^, respectively. Participants who spent more time on specific academic study-related or screen-related sedentary behaviors had significantly higher BMI. More frequent SSB consumption was significantly associated with higher BMI (β (95% CI) for <1 time/day and 1+ time/day: 0.30 (0.17, 0.42) and 0.58 (0.41, 0.76) kg/m^2^) (Table 3).

### 3.3. Mutually Adjusted Results and the Importance Ranking of Five Specific Sedentary Behaviors

After additionally adjusted for SSB consumption, longer screen-related, academic study-related and total sedentary time were associated with a higher risk of overweight/obesity (RR (95% CI) per hour increment: 1.01 (1.00–1.02), 1.03 (1.01–1.06) and 1.02 (1.01–1.03), respectively) and higher BMI (β (95% CI) per hour increment: 0.02 (0.01, 0.03), 0.11 (0.10, 0.13) and 0.04 (0.03, 0.05) kg/m^2^, respectively) (Table 2 and Table 3). Correlation coefficients among variables included in the mutually adjusted model for specific sedentary behaviors were from −0.359 to 0.366 (Figure S2). With mutual adjustments, participants who spent more time on homework, attending tutorial classes, and using mobile electronic devices had significantly higher overweight/obesity incidence and higher BMI, whereas more time spent on using a computer was significantly associated with higher BMI only (β (95% CI) for 1+ hour/day: 0.10 (0.00, 0.19) kg/m^2^). Compared with those who never watched TV, participants who spent less than 1 h/day on watching TV had significantly lower BMI (β (95% CI): −0.12 (−0.20, −0.03) kg/m^2^) (Table 3).

**Figure 1:**
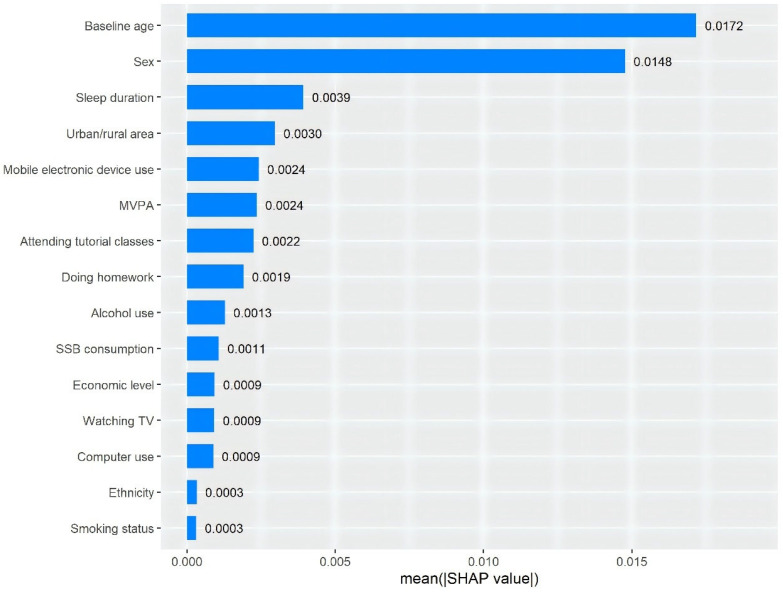
Importance matrix and SHAP summary plot displaying the contributions of individual variables to the extreme Gradient Boosting (XGBoost) model. SHAP = Shapley additive explanations, MVPA = moderate-to-vigorous physical activity, SSB = sugar-sweetened beverage.

Based on the SHAP plot from the XGBoost algorithm model, in the full adjustment model, mobile electronic device use appeared to be the most important sedentary factor contributing to overweight/obesity incidence, while attending tutorial classes and doing homework ranked second and third, respectively (Figure 1).

### 3.4. Joint Associations of Total Sedentary Time and SSB Consumption with Overweight/Obesity Incidence and BMI

No significant interactions were found between total sedentary time and SSB consumption on overweight/obesity incidence or BMI (*p* for interaction: 0.234 and 0.239). Compared with those who never drank SSB and had short sedentary time, participants who drank SSB of <1 time/day and had long sedentary time had a significantly higher risk of overweight/obesity (RR (95% CI): 1.22 (1.08–1.37)). Moreover, with “never drink & short sedentary time” as the reference group, more frequent SSB consumption and longer sedentary time were significantly associated with higher BMI (β (95% CI) for “never drink & long sedentary time”, “<1 time/day & short sedentary time”, “<1 time/day & long sedentary time”, “1+ time/day & short sedentary time”, “1+ time/day & long sedentary time” group: 0.25 (0.11, 0.40), 0.18 (0.09, 0.28), 0.42 (0.32, 0.52), 0.45 (0.25, 0.65) and 0.49 (0.35, 0.64) kg/m^2^, respectively). (Figure S3 and Table S2).

### 3.5. Sensitivity Analyses

Significant interactions were found for screen-related, academic study-related and total sedentary time with age on overweight/obesity incidence or BMI (*p* for interaction < 0.05). The associations of screen-related or total sedentary time with overweight/obesity incidence or BMI were more pronounced in participants aged below 12 years, while results of academic study-related sedentary time were more evident in participants aged 12+ years. Age-stratified analyses showed similar results when age 10 was used as the stratification threshold (Table S3). Significant interactions were also found between screen-related sedentary time and sex on BMI, with this association being more evident in women (*p* for interaction = 0.042) (Table 4). When separating overweight incidence and obesity incidence, in the mutually adjusted model, longer total, screen-related, and academic study-related sedentary time, and higher SSB consumption were associated with higher obesity incidence (Table S4). Participants with longer academic study-related sedentary time and more time spent attending tutorial classes or using mobile electronic devices were significantly associated with higher overweight incidence (Table S5). The associations of sedentary behaviors and SSB consumption with overweight/obesity incidence redefined by IOTF criteria showed results similar to those in the main analyses (Tables S2 and S6). In addition, participants included and excluded in our analysis were comparable, with Cohen’s effect sizes ranging from 0.01 to 0.13 (Table S7).

## 4. Discussion

In this large population-based longitudinal analysis in participants aged 6–20 years, longer academic study-related, screen-related, and total sedentary time were associated with a higher risk of overweight/obesity and higher levels of BMI. Among the five specific sedentary behaviors, mobile electronic device use, attending tutorial classes, and doing homework were the three most important sedentary behaviors contributing to incident overweight/obesity. No significant interactions between SSB consumption and sedentary time on overweight/obesity incidence or BMI were found. Besides mobile electronic device use, our results underscore the importance of addressing academic study-related sedentary behaviors in obesity prevention strategies. Since academic study-related behaviors are largely overlooked, our findings highlight the need for targeted public health interventions to reduce mobile electronic device use and academic study-related sedentary behaviors to prevent obesity in children and adolescents.

One of the important innovations in our study was the use of the SHAP plot to generate interpretable rankings of the relative contributions of specific sedentary behaviors to childhood and adolescent obesity. Based on our SHAP plot’s importance assessment, mobile electronic device use, attending tutorial classes, and doing homework were the three most important sedentary factors contributing to incident overweight/obesity, suggesting the need for targeted prevention strategies. Given the increasing use of mobile electronic devices among children and adolescents, it should be a focus for prevention efforts [30]. Furthermore, in settings with competitive educational systems, such as China [31], it is crucial to evaluate the impact of academic study-related sedentary behaviors on obesity. Most previous studies showed a positive association between doing homework and obesity [32,33,34,35,36] while two studies showed no significant association, probably due to the small sample size [37] or minor increment in the continuous variable per unit (min/day) [38]. Moreover, there are limited studies reporting the association between attending tutorial classes and obesity, with limitations such as lack of longitudinal design or small sample sizes [32,33]. Given the longitudinal design and large, representative study sample, our findings provide strong evidence for the primarily adverse role of doing homework and attending tutorial classes in obesity, and highlight the importance of reducing these academic study-related sedentary behaviors in childhood and adolescent obesity prevention.

Our findings that longer total and screen-related sedentary time were associated with higher overweight/obesity risk and higher BMI were consistent with previous studies. For example, a 2020 systematic review of 17 studies conducted in China showed that sedentary behavior, especially screen-related sedentary behavior, was associated with a higher risk of obesity or higher BMI in Chinese children aged 6–18 years [9]. Most studies published after this review also showed a positive association between sedentary behavior and obesity [38,39,40,41,42,43,44] with potential explanations including reduced energy expenditure [45] or related unhealthy dietary patterns [15]. Our study adds to this body of literature by showing that longer academic study-related sedentary time was also associated with a higher risk of overweight/obesity and higher BMI, given few studies specifically assessed the association between academic study-related sedentary time and obesity [46,47]. A longitudinal study involving 2228 participants aged 6–19 years in China found no significant association between educational screen time and obesity risk [46]. This finding may be attributed to the exclusive focus on educational screen time including online homework and class activities, while neglecting other sedentary behaviors associated with studying, such as paper-based assignments or attending tutorial sessions [46]. Consequently, this study might not fully capture the impact of academic study-related sedentary behavior on obesity risk. Another cross-sectional study of 4852 adolescents aged 11–19 years showed that accelerometer measured school-based sedentary time was associated with lower odds of overweight/obesity and lower BMI [47]. The authors explained that this could be due to a compensation mechanism from vigorous physical activity (VPA), where participants with active VPA may spend more time sitting, which could be captured by accelerometer measurement [47]. However, our study also accounted for physical activity and found that academic study-related sedentary behaviors still had significant association with overweight/obesity.

Furthermore, our study found that the effect sizes of academic study-related sedentary time on overweight/obesity risk or BMI were larger than those of screen-related sedentary time. One possible explanation is that academic study-related behaviors such as doing homework or attending tutorial classes are linked to higher psychological stress, which is a known risk factor for obesity [2]. Another explanation is that parents may reward children with junk food to increase compliance with homework and tutoring, which, combined with prolonged sitting and unhealthy food intake, further increases obesity risk [48].

Another finding of our study was that the associations of sedentary time with overweight/obesity risk or BMI varied by sex or age. First, we found that the adverse association between screen-related sedentary time and BMI was more pronounced in women than in men, which was consistent with the result from a cross-sectional study conducted in China [49] and likely attributable to difference in body composition between men and women [50]. Second, in our age-stratified analysis, the positive association of screen-related and total sedentary time with overweight/obesity risk and BMI were more evident in participants aged below 12 years. This could be because children aged 5–7 years were in the period of adiposity rebound, making them more sensitive to obesogenic factors such as sedentary and screening behaviors, thus increasing the likelihood of becoming obese [51]. In contrast, the positive association between academic study-related sedentary time and overweight/obesity risk or BMI were more pronounced in participants aged 12+ years, potential due to higher academic stress in this age group. A previous study reported that the association of doing homework with obesity indicators varied by schoolwork-related stress, with the adverse association being observed only in participants stressed from schoolwork [36]. Therefore, since high school students (generally aged 12+ years) experienced higher schoolwork-related stress than primary school students, the detrimental influence of academic study-related sedentary time may be more pronounced in this older age group. Furthermore, acknowledging concerns about recall accuracy among younger children, we performed stratified analyses using age 10 as a cut-off. While the overall patterns were consistent across age groups, the potential for less reliable self-reported sedentary behavior data among children under 10 years warrants cautious interpretation of these subgroup findings. Regarding the joint associations, our study found no significant interactions between sedentary time and SSB consumption on overweight/obesity risk or BMI, indicating that their coexistence did not have a synergistic effect on the occurrence of obesity.

Our findings provide novel scientific evidence to support targeted interventions to prevent childhood and adolescent obesity. Parents should monitor and regulate academic study-related sedentary time, ensuring children have balanced routines that include regular physical activity. Schools should incorporate structured physical activity breaks to mitigate these effects. Policies should enforce comprehensive physical activity programs in schools and implement strategies to reduce academic demands, fostering healthier lifestyles for children and adolescents.

The strengths of our study included the large and representative study sample, longitudinal design, comprehensive exploration of total and specific types of sedentary behaviors, and categorical and continuous obesity-related outcomes. Additionally, the SHAP plot’s importance assessment allowed us to detect the prominent obesogenic sedentary behaviors. However, our study also had some limitations. First, SSB consumption and sedentary behavior data were collected via self-reported questionnaire, which are subject to measurement error, despite efforts to help participants accurately recall specific behaviors. Second, although we categorized mobile electronic device use as screen-related sedentary behavior in this paper, these devices, such as iPads, could also be used for learning purposes. Therefore, it was difficult to clearly differentiate the effects of academic study- and non-study-related sedentary behaviors in it. Third, our study focused solely on the frequency of SSB consumption without considering the actual quantity consumed. Future studies with more comprehensive data collection on SSB consumption are warranted. Fourth, the absence of waist circumference measurements limited our ability to evaluate the association between SSB consumption and central obesity, suggesting that further investigations should incorporate more detailed anthropometric parameters to enhance the understanding of this association. Fifth, the follow-up duration in this study was relatively short, which may limit the ability to capture long-term changes in weight status. Nevertheless, given the large sample size and the relatively high incidence of overweight and obesity during this period, we believe that the study duration was sufficient to detect meaningful associations. Further studies with longer follow-up periods are needed to confirm these findings and explore long-term trajectories. Finally, despite adjusting for multiple potential confounders, residual confounding from unmeasured factors such as daily energy intake or psychological stress could not be ruled out, and thus we could not establish causality in this study.

## 5. Conclusions

Excessive academic study- and non-study-related sedentary behaviors were associated with a higher risk of overweight/obesity, with using mobile electronic devices, attending tutorial classes and doing homework being the three most important sedentary factors contributing to overweight/obesity. No significant interaction between total sedentary time and SSB consumption was found. These findings highlight the need for targeted public health interventions to reduce mobile electronic device use and academic study-related sedentary behaviors, and promote a balanced lifestyle among children and adolescents.

## Figures and Tables

**Table 1:** Baseline characteristics of 47,148 participants aged 6–20 years in Guangdong province (2019–2021), stratified by overweight/obesity status at follow-up in 2022.

	Overall	Follow-Up Overweight/Obesity Status		*p* Value
		No	Yes	
Number of participants, *N* (%)	47,148 (100.00)	44,197 (93.74)	2951 (6.26)	-
Age, years, mean (SD)	12.39 (2.86)	12.44 (2.84)	11.62 (2.98)	<0.001
Sex, %				<0.001
Men	49.20	48.29	62.83	
Women	50.80	51.71	37.17	
Ethnicity, %				0.559
Han	98.17	98.18	98.03	
Others	1.83	1.82	1.97	
Area, %				<0.001
Urban	55.04	54.81	58.52	
Rural	44.96	45.19	41.48	
Economic level, %				0.475
Low	31.44	31.38	32.29	
Middle	31.60	31.60	31.68	
High	36.96	37.02	36.02	
Smoking status, %				0.411
Never	95.34	95.35	95.15	
Former	3.98	3.95	4.31	
Current	0.68	0.69	0.54	
Alcohol use, %				0.561
No	81.75	81.78	81.35	
Yes	18.25	18.22	18.65	
MVPA frequency, day/week				<0.001
0–1	29.35	29.57	25.95	
2–3	34.31	34.29	34.53	
4–5	18.20	18.18	18.49	
6–7	18.15	17.95	21.03	
Diabetes, %				0.891
No	99.97	99.97	99.97	
Yes	0.03	0.03	0.03	
Hypertension, %				0.073
No	99.98	99.98	99.93	
Yes	0.02	0.02	0.07	
Cardiovascular disease, %				0.592
No	99.90	99.90	99.93	
Yes	0.10	0.10	0.07	
Sleep duration, hour/day, mean (SD)	8.27 (1.83)	8.27 (1.82)	8.36 (1.90)	0.010
BMI, kg/m^2^, mean (SD)	17.28 (2.37)	17.16 (2.32)	19.00 (2.48)	<0.001

*N* = number, SD = standard deviation, BMI = body mass index, MVPA = moderate-to-vigorous physical activity.

**Table 2:** Associations of baseline sedentary behaviors and sugar-sweetened beverage consumption with overweight/obesity incidence in participants aged 6–20 years in Guangdong province in 2019–2021 and followed up in 2022.

	Participants with Overweight/Obesity, *N* (%)	Overweight/Obesity, RR (95% CI)		
		Crude Model	Model 1	Model 2
Doing homework (x), hour/day				
0 ≤ x < 1	635 (6.59)	1.00	1.00	1.00
1 ≤ x < 2	1198 (6.74)	1.02 (0.93, 1.12)	1.10 (1.01, 1.21) *	1.10 (1.00, 1.21)
2 ≤ x < 3	689 (5.73)	0.87 (0.78, 0.97) **	1.04 (0.94, 1.16)	1.04 (0.93, 1.16)
x ≥ 3	549 (5.79)	0.88 (0.79, 0.98) *	1.14 (1.02, 1.28) *	1.13 (1.00, 1.27) *
Attending tutorial classes (x), hour/week				
0 ≤ x < 1	1998 (5.90)	1.00	1.00	1.00
1 ≤ x < 2	458 (7.15)	1.21 (1.10, 1.34) ***	1.08 (0.98, 1.20)	1.08 (0.97, 1.19)
2 ≤ x < 3	303 (6.77)	1.15 (1.02, 1.29) *	1.04 (0.92, 1.17)	1.04 (0.93, 1.18)
x ≥ 3	351 (7.30)	1.24 (1.11, 1.38) ***	1.17 (1.05, 1.31) **	1.16 (1.04, 1.31) *
Watching TV (x), hour/day				
0	554 (5.91)	1.00	1.00	1.00
0 < x < 1	1332 (6.37)	1.08 (0.98, 1.19)	1.01 (0.92, 1.11)	0.99 (0.89, 1.09)
1 ≤ x < 2	771 (6.21)	1.05 (0.94, 1.17)	0.99 (0.89, 1.10)	0.93 (0.83, 1.05)
x ≥ 2	567 (6.48)	1.10 (0.98, 1.23)	1.04 (0.93, 1.17)	0.97 (0.85, 1.10)
Computer use (x), hour/day				
0	1466 (6.09)	1.00	1.00	1.00
0 < x < 1	1034 (6.28)	1.03 (0.96, 1.11)	1.05 (0.97, 1.13)	1.03 (0.95, 1.12)
1 ≤ x < 2	408 (6.69)	1.10 (0.99, 1.22)	1.11 (1.00, 1.24)	1.04 (0.92, 1.17)
x ≥ 2	317 (6.57)	1.08 (0.96, 1.21)	1.06 (0.94, 1.20)	0.99 (0.87, 1.13)
Mobile electronic device use (x), hour/day				
0	767 (6.36)	1.00	1.00	1.00
0 < x < 1	685 (6.35)	1.00 (0.90, 1.10)	1.02 (0.92, 1.13)	1.00 (0.90, 1.11)
1 ≤ x < 2	856 (6.47)	1.02 (0.93, 1.12)	1.09 (0.99, 1.20)	1.08 (0.97, 1.20)
x ≥ 2	908 (5.96)	0.94 (0.85, 1.03)	1.20 (1.08, 1.33) ***	1.20 (1.07, 1.34) **
Screen-related sedentary time, hour/day				
Per hour increment	3197 (6.25)	1.00 (0.99, 1.01)	1.01 (1.00, 1.03) *	1.01 (1.00, 1.02) *
Academic study-related sedentary time, hour/day				
Per hour increment	2979 (6.27)	1.02 (0.99, 1.04)	1.03 (1.01, 1.06) **	1.03 (1.01, 1.06) **
Total sedentary time, hour/day				
Per hour increment	2951 (6.26)	0.99 (0.98, 1.01)	1.02 (1.01, 1.03) **	1.02 (1.01, 1.03) **
SSB consumption (x), time/day				
0	571 (6.18)	1.00	1.00	1.00
0 < x < 1	2351 (6.23)	1.01 (0.92, 1.10)	1.09 (0.93, 1.29)	1.10 (0.93, 1.30)
x ≥ 1	300 (6.70)	1.08 (0.95, 1.24)	1.23 (0.98, 1.53)	1.11 (0.88, 1.41)

*N* = number, RR = relative risk, CI = confidence interval, SSB = sugar-sweetened beverage. Model 1: adjusted for sex, baseline age, ethnicity, urban/rural area, economic level, smoking status, alcohol use, moderate-to-vigorous physical activity, sleep duration, chronic disease history (diabetes, hypertension and cardiovascular disease), and food groups (dessert, fried food, fruit, and vegetable) consumption (for SSB consumption only). Model 2: mutually adjusted for total sedentary time and SSB consumption (for SSB consumption and total or specific sedentary time). For specific sedentary behaviors, Model 2 was additionally mutually adjusted for specific sedentary behaviors. * *p* < 0.05, ** *p* < 0.01, *** *p* < 0.001.

**Table 3:** Associations of baseline sedentary behaviors and sugar-sweetened beverage consumption with followed-up body mass index (BMI) in participants aged 6–20 years in Guangdong province in 2019–2021 and followed up in 2022.

	Number of Participants, *N* (%)	BMI, kg/m^2^, β (95% CI)		
		Crude Model	Model 1	Model 2
Doing homework (x), hour/day				
0 ≤ x < 1	11,765 (19.60)	0.00	0.00	0.00
1 ≤ x < 2	21,902 (36.48)	0.44 (0.36, 0.52) ***	0.20 (0.12, 0.27) ***	0.17 (0.09, 0.25) ***
2 ≤ x < 3	14,829 (24.70)	0.98 (0.89, 1.07) ***	0.35 (0.27, 0.44) ***	0.29 (0.20, 0.38) ***
x ≥ 3	11,539 (19.22)	1.28 (1.19, 1.38) ***	0.35 (0.25, 0.44) ***	0.27 (0.18, 0.37) ***
Attending tutorial classes (x), hour/week				
0 ≤ x < 1	40,924 (67.31)	0.00	0.00	0.00
1 ≤ x < 2	7988 (13.14)	−0.24 (−0.33, −0.15) ***	0.12 (0.03, 0.20) **	0.11 (0.03, 0.20) *
2 ≤ x < 3	5693 (9.36)	0.01 (−0.09, 0.11)	0.24 (0.14, 0.33) ***	0.19 (0.09, 0.29) ***
x ≥ 3	6194 (10.19)	0.50 (0.40, 0.60) ***	0.43 (0.34, 0.53) ***	0.39 (0.29, 0.49) ***
Watching TV (x), hour/day				
0	11,432 (18.12)	0.00	0.00	0.00
0 < x < 1	25,281 (40.07)	−0.46 (−0.54, −0.38) ***	−0.07 (−0.15, 0.01)	−0.12 (−0.20, −0.03) **
1 ≤ x < 2	15,383 (24.38)	−0.15 (−0.24, −0.06) **	0.06 (−0.03, 0.14)	−0.03 (−0.12, 0.06)
x ≥ 2	11,000 (17.43)	0.12 (0.02, 0.21) *	0.18 (0.08, 0.27) ***	0.06 (−0.05, 0.16)
Computer use (x), hour/day				
0	29,311 (46.45)	0.00	0.00	0.00
0 < x < 1	20,059 (31.79)	0.25 (0.19, 0.32) ***	0.02 (−0.04, 0.08)	0.01 (−0.05, 0.08)
1 ≤ x < 2	7614 (12.07)	0.66 (0.56, 0.75) ***	0.17 (0.08, 0.26) ***	0.10 (0.00, 0.19) *
x ≥ 2	6118 (9.70)	0.71 (0.61, 0.81) ***	0.20 (0.10, 0.30) ***	0.09 (−0.02, 0.20)
Mobile electronic device use (x), hour/day				
0	14,672 (23.32)	0.00	0.00	0.00
0 < x < 1	13,279 (21.10)	0.29 (0.21, 0.38) ***	0.10 (0.02, 0.19) *	0.08 (−0.01, 0.17)
1 ≤ x < 2	16,317 (25.93)	0.56 (0.47, 0.64) ***	0.18 (0.10, 0.26) ***	0.14 (0.05, 0.23) **
x ≥ 2	18,653 (29.65)	1.35 (1.27, 1.42) ***	0.23 (0.15, 0.31) ***	0.14 (0.05, 0.23) **
Screen-related sedentary time, hour/day				
Per hour increment	62,702 (100.00)	0.13 (0.12, 0.14) ***	0.03 (0.02, 0.04) ***	0.02 (0.01, 0.03) ***
Academic study-related sedentary time, hour/day				
Per hour increment	58,318 (100.00)	0.22 (0.20, 0.24) ***	0.12 (0.10, 0.14) ***	0.11 (0.10, 0.13) ***
Total sedentary time, hour/day				
Per hour increment	57,885 (100.00)	0.17 (0.16, 0.18) ***	0.04 (0.03, 0.05) ***	0.04 (0.03, 0.05) ***
SSB consumption (x), time/day				
0	11,155 (17.68)	0.00	0.00	0.00
0 < x < 1	46,288 (73.36)	0.76 (0.69, 0.84) ***	0.30 (0.17, 0.42) ***	0.26 (0.13, 0.39) ***
x ≥ 1	5652 (8.96)	1.27 (1.15, 1.38) ***	0.58 (0.41, 0.76) ***	0.51 (0.32, 0.69) ***

*N* = number, CI = confidence interval, BMI = body mass index, SSB = sugar-sweetened beverage. Model 1: adjusted for sex, baseline age, ethnicity, urban/rural area, economic level, smoking status, alcohol use, moderate-to-vigorous physical activity, sleep duration, chronic disease history (diabetes, hypertension and cardiovascular disease), and food groups (dessert, fried food, fruit, and vegetable) consumption (for SSB consumption only). Model 2: mutually adjusted for total sedentary time and SSB consumption (for SSB consumption and total or specific sedentary time). For specific sedentary behaviors, Model 2 was additionally mutually adjusted for specific sedentary behaviors. * *p* < 0.05, ** *p* < 0.01, *** *p* < 0.001.

**Table 4:** Associations of baseline sedentary behaviors and sugar-sweetened beverage consumption with overweight/obesity incidence or follow-up body mass index (BMI) in participants aged 6–20 years in Guangdong province in 2019–2021 and followed up in 2022, stratified by sex or age groups.

	Sex		*p* for Interaction	Age		*p* for Interaction
	Men	Women		<12 Years	12+ Years	
**Overweight/obesity status, RR (95% CI) ^a^**						
Screen-related sedentary time, hour/day			0.063			0.004
Per hour increment	1.01 (0.99, 1.02)	1.03 (1.01, 1.05) **		1.03 (1.01, 1.05) ***	0.99 (0.98, 1.01)	
Academic study-related sedentary time, hour/day			0.341			<0.001
Per hour increment	1.03 (1.00, 1.06)	1.05 (1.01, 1.09) *		0.99 (0.96, 1.02)	1.09 (1.05, 1.13) ***	
Total sedentary time, hour/day			0.085			0.030
Per hour increment	1.01 (1.00, 1.03)	1.03 (1.01, 1.05) **		1.03 (1.01, 1.05) **	1.00 (0.98, 1.02)	
SSB consumption (x), time/day			0.351			0.516
0	1.00	1.00		1.00	1.00	
0 < x < 1	1.10 (0.90, 1.36)	1.07 (0.82, 1.39)		0.92 (0.73, 1.15)	1.28 (1.01, 1.62) *	
x ≥ 1	1.10 (0.84, 1.45)	1.48 (1.02, 2.13) *		0.99 (0.69, 1.41)	1.46 (1.09, 1.96) *	
**BMI, kg/m^2^, β (95% CI) ^b^**						
Screen-related sedentary time, hour/day			0.042			<0.001
Per hour increment	0.02 (0.01, 0.03) **	0.04 (0.02, 0.05) ***		0.08 (0.07, 0.10) ***	0.02 (0.01, 0.03) **	
Academic study-related sedentary time, hour/day			0.199			0.009
Per hour increment	0.12 (0.09, 0.15) ***	0.11 (0.08, 0.13) ***		0.08 (0.05, 0.11) ***	0.12 (0.10, 0.15) ***	
Total sedentary time, hour/day			0.087			<0.001
Per hour increment	0.03 (0.02, 0.05) ***	0.05 (0.04, 0.06) ***		0.09 (0.08, 0.11) ***	0.03 (0.02, 0.04) ***	
SSB consumption (x), time/day			0.651			0.808
0	0.00	0.00		0.00	0.00	
0 < x < 1	0.36 (0.17, 0.55) ***	0.22 (0.07, 0.38) **		0.26 (0.06, 0.45) *	0.28 (0.12, 0.44) **	
x ≥ 1	0.66 (0.41, 0.92) ***	0.49 (0.26, 0.72) ***		0.39 (0.07, 0.72) *	0.63 (0.42, 0.85) ***	

RR = relative risk, BMI = body mass index, CI = confidence interval, SSB = sugar-sweetened beverage. ^a^ RRs (95% CIs) were adjusted for sex, baseline age, ethnicity, urban/rural area, economic level, smoking status, alcohol use, moderate-to-vigorous physical activity, sleep duration, chronic disease history (diabetes, hypertension and cardiovascular disease), and food groups (dessert, fried food, fruit, and vegetable) consumption (for SSB consumption only), as appropriate. ^b^ βs (95% CIs) were adjusted for sex, baseline age, ethnicity, urban/rural area, economic level, smoking status, alcohol use, moderate-to-vigorous physical activity, sleep duration, chronic disease history (diabetes, hypertension and cardiovascular disease), and food groups (dessert, fried food, fruit, and vegetable) consumption (for SSB consumption only), as appropriate. * *p* < 0.05, ** *p* < 0.01, *** *p* < 0.001.

## Data Availability

Deidentified individual participant data will not be made available.

## Supplementary Materials

The following supporting information can be downloaded at: https://www.mdpi.com/article/10.3390/nu17101633/s1, Table S1. Age- and sex-specific body mass index cut-off values (kg/m^2^) according to the criteria of “Screening for overweight and obesity among school-age children and adolescents” in China. Table S2. Joint associations of total sedentary time and sugar-sweetened beverage consumption with overweight and/or obesity incidence, and followed-up body mass index (BMI) in participants aged 6–20 years in Guangdong province in 2019–2021 and followed up till 2022. Table S3. Associations of baseline sedentary behaviors and sugar-sweetened beverage consumption with overweight/obesity incidence or follow-up body mass index (BMI) in participants aged 6–20 years in Guangdong province in 2019–2021 and followed up till 2022 stratified by age groups (<10 years vs. ≥10 years). Table S4. Associations of baseline sedentary behaviors and sugar-sweetened beverage consumption with obesity incidence in participants aged 6–20 years in Guangdong province in 2019–2021 and followed up till 2022. Table S5. Associations of baseline sedentary behaviors and sugar-sweetened beverage consumption with overweight incidence in participants aged 6–20 years in Guangdong province in 2019–2021 and followed up till 2022. Table S6. Associations of baseline sedentary behaviors and sugar-sweetened beverage consumption with overweight/obesity incidence ^a^ in participants aged 6–20 years in Guangdong province in 2019–2021 and followed up till 2022. Table S7. Comparison of baseline characteristics between participants included and not included in the main analysis of the associations between total sedentary time and overweight/obesity incidence. Figure S1. Flow chart of surveillance design and participants selection in the main analysis. Figure S2. Heatmap of correlation coefficients among variables included in the mutually adjusted model for specific sedentary behaviors. Figure S3. Adjusted relative risks/βs ^a^ for overweight/obesity incidence and followed-up body mass index (BMI) by groups of total sedentary time and sugar-sweetened beverage consumption in participants aged 6–20 years in Guangdong province in 2019–2021 and followed up till 2022.
