# Supplementary material for: Associations of Academic Study- and Non-Study-Related Sedentary Behaviors with Incident Obesity in Children and Adolescents
Source: Nutrients. 2025 May 9;17(10):1633. doi: 10.3390/nu17101633 (PMC12114288; doi:10.3390/nu17101633)
Supplement: Supplementary file 1 [file nutrients-17-01633-s001.zip › nutrients-3617561-supplementary.pdf]

Associations of academic study- and non-study-related sedentary behaviors with incident obesity in children and adolescents

Contents

Table S1 Age- and sex-specific body mass index cut-off values (kg/m^2) according to the criteria of "Screening for overweight and obesity among school-age children and adolescents" in China

Table S2 Joint associations of total sedentary time and sugar-sweetened beverage consumption with overweight and/or obesity incidence, and followed-up body mass index (BMI) in participants aged 6-20 years in Guangdong province in 2019-2021 and followed up till 2022

Table S3 Associations of baseline sedentary behaviors and sugar-sweetened beverage consumption with overweight/obesity incidence or follow-up body mass index (BMI) in participants aged 6-20 years in Guangdong province in 2019-2021 and followed up till 2022 stratified by age groups (<10 years vs ≥10 years)

Table S4 Associations of baseline sedentary behaviors and sugar-sweetened beverage consumption with obesity incidence in participants aged 6-20 years in Guangdong province in 2019-2021 and followed up till 2022

Table S5 Associations of baseline sedentary behaviors and sugar-sweetened beverage consumption with overweight incidence in participants aged 6-20 years in Guangdong province in 2019-2021 and followed up till 2022

Table S6 Associations of baseline sedentary behaviors and sugar-sweetened beverage

consumption with overweight/obesity incidence [a] in participants aged 6-20 years in Guangdong province in 2019-2021 and followed up till 2022

Table S7 Comparison of baseline characteristics between participants included and not included in the main analysis of the associations between total sedentary time and overweight/obesity incidence

Figure S1 Flow chart of surveillance design and participants selection in the main analysis

Figure S2 Heatmap of correlation coefficients among variables included in the mutually adjusted model for specific sedentary behaviors

Figure S3 Adjusted relative risks/βs [a] for overweight/obesity incidence and followed-up body mass index (BMI) by groups of total sedentary time and sugar-sweetened beverage consumption in participants aged 6-20 years in Guangdong province in 2019-2021 and followed up till 2022

Table S1 Age- and sex-specific body mass index cut-off values (kg/m^2) according to the criteria of "Screening for overweight and obesity among school-age children and adolescents" in China

Age (years)	Men		Women	
	Overweight	Obesity	Overweight	Obesity
6.0+	16.4	17.7	16.2	17.5
6.5+	16.7	18.1	16.5	18.0
7.0+	17.0	18.7	16.8	18.5
7.5+	17.4	19.2	17.2	19.0
8.0+	17.8	19.7	17.6	19.4
8.5+	18.1	20.3	18.1	19.9
9.0+	18.5	20.8	18.5	20.4
9.5+	18.9	21.4	19.0	21.0
10.0+	19.2	21.9	19.5	21.5
10.5+	19.6	22.5	20.0	22.1
11.0+	19.9	23.0	20.5	22.7
11.5+	20.3	23.6	21.1	23.3
12.0+	20.7	24.1	21.5	23.9
12.5+	21.0	24.7	21.9	24.5
13.0+	21.4	25.2	22.2	25.0
13.5+	21.9	25.7	22.6	25.6
14.0+	22.3	26.1	22.8	25.9
14.5+	22.6	26.4	23.0	26.3
15.0+	22.9	26.6	23.2	26.6
15.5+	23.1	26.9	23.4	26.9
16.0+	23.3	27.1	23.6	27.1
16.5+	23.5	27.4	23.7	27.4
17.0+	23.7	27.6	23.8	27.6
17.5+	23.8	27.8	23.9	27.8
18.0+	24.0	28.0	24.0	28.0

Table S2 Joint associations of total sedentary time and sugar-sweetened beverage consumption with overweight and/or obesity incidence, and followed-up body mass index (BMI) in participants aged 6-20 years in Guangdong province in 2019-2021 and followed up till 2022

	Total sedentary time and SSB consumption					
	**Never drink & short sedentary time**	**Never drink & long sedentary time**	**<1 time/day & short sedentary time**	**<1 time/day & long sedentary time**	**1+ time/day & short sedentary time**	**1+ time/day & long sedentary time**
Overweight/obesity [a], RR (95% CI)						
Crude model	1.00	0.98 (0.82, 1.16)	1.00 (0.89, 1.13)	0.98 (0.88, 1.10)	1.13 (0.90, 1.44)	0.94 (0.79, 1.12)
Model 1	1.00	1.13 (0.95, 1.35)	1.07 (0.95, 1.20)	1.22 (1.08, 1.37) **	1.24 (0.98, 1.58)	1.10 (0.92, 1.32)
BMI, kg/m^2, β (95% CI)						
Crude model	0.00	0.93 (0.78, 1.08) ***	0.56 (0.46, 0.66) ***	1.49 (1.39, 1.58) ***	1.21 (1.01, 1.42) ***	1.76 (1.61, 1.91) ***
Model 1	0.00	0.25 (0.11, 0.40) **	0.18 (0.09, 0.28) ***	0.42 (0.32, 0.52) ***	0.45 (0.25, 0.65) ***	0.49 (0.35, 0.64) ***
Obesity [a], RR (95% CI)						
Crude model	1.00	1.24 (0.95, 1.61)	1.07 (0.89, 1.29)	1.07 (0.89, 1.28)	1.27 (0.89, 1.82)	1.24 (0.96, 1.61)
Model 1	1.00	1.48 (1.13, 1.93) **	1.17 (0.97, 1.41)	1.45 (1.19, 1.76) ***	1.41 (0.97, 2.03)	1.62 (1.24, 2.13) ***
Overweight [a], RR (95% CI)						
Crude model	1.00	0.96 (0.80, 1.17)	1.02 (0.91, 1.16)	1.01 (0.90, 1.14)	1.20 (0.94, 1.54)	0.95 (0.79, 1.14)
Model 1	1.00	1.10 (0.91, 1.33)	1.07 (0.95, 1.21)	1.20 (1.06, 1.36) **	1.28 (0.99, 1.63)	1.05 (0.87, 1.28)
Overweight/obesity [b], RR (95% CI)						
Crude model	1.00	0.99 (0.83, 1.19)	1.00 (0.89, 1.12)	0.89 (0.79, 1.00)	1.07 (0.83, 1.37)	0.90 (0.75, 1.08)
Model 1	1.00	1.24 (1.04, 1.48) *	1.11 (0.99, 1.25)	1.26 (1.11, 1.42) ***	1.26 (0.98, 1.62)	1.23 (1.02, 1.49) *

RR=relative risk, CI=confidence interval, SSB=sugar-sweetened beverage, BMI=body mass index.

[a] Overweight/obesity was defined by the age- and sex-specific BMI cut-off values according to the criteria of "Screening for overweight and obesity among school-age children and adolescents" in China.

[b] Overweight/obesity was defined by the age- and sex-specific BMI cut-off values from International Obesity Task Force (IOTF).

Model 1: adjusted for sex, baseline age, ethnicity, urban/rural area, economic level, smoking status, alcohol use, moderate-to-vigorous physical activity, sleep duration and chronic disease history (diabetes, hypertension and cardiovascular disease).

$^{*}P<0.05$, $^{**}P<0.01$, $^{***}P<0.001$.

Table S3 Associations of baseline sedentary behaviors and sugar-sweetened beverage consumption with overweight/obesity incidence or follow-up body mass index (BMI) in participants aged 6-20 years in Guangdong province in 2019-2021 and followed up till 2022 stratified by age groups (<10 years vs ≥10 years)

	**Age<10 year**	**Age≥10 years**
	**Overweight/obesity status, RR (95% CI) [a]**	
Screen-related sedentary time, hour/day		
Per hour increment	1.04 (1.02, 1.06) ***	0.99 (0.98, 1.01)
Academic study-related sedentary time, hour/day		
Per hour increment	0.99 (0.96, 1.03)	1.07 (1.03, 1.10) ***
Total sedentary time, hour/day		
Per hour increment	1.07 (1.03, 1.10) ***	1.04 (1.02, 1.06) ***
SSB consumption (x), time/day		
0	1.00	1.00
0<x<1	1.02 (0.72, 1.45)	1.11 (0.93, 1.34)
x≥1	0.94 (0.54, 1.66)	1.27 (0.99, 1.62)
	**BMI, kg/m^2, β (95% CI) [b]**	
Screen-related sedentary time, hour/day		
Per hour increment	0.09 (0.07, 0.11) ***	0.05 (0.04, 0.06) ***
Academic study-related sedentary time, hour/day		
Per hour increment	0.07 (0.04, 0.11) ***	0.12 (0.10, 0.15) ***
Total sedentary time, hour/day		
Per hour increment	0.09 (0.07, 0.11) ***	0.07 (0.06, 0.08) ***
SSB consumption (x), time/day		
0	0.00	0.00
0<x<1	0.16 (-0.14, 0.45)	0.32 (0.18, 0.46) ***
x≥1	0.34 (-0.16, 0.84)	0.69 (0.50, 0.88) ***

RR=relative risk, BMI=body mass index, CI=confidence interval, SSB=sugar-sweetened beverage.

[a] RRs (95% CIs) were adjusted for sex, ethnicity, urban/rural area, economic level, smoking status, alcohol use, moderate-to-vigorous physical activity, sleep duration, chronic disease history (diabetes, hypertension and cardiovascular disease) and food groups (dessert, fried food, fruit and vegetable) consumption (for SSB consumption only).

[b] βs (95% CIs) were adjusted for sex, ethnicity, urban/rural area, economic level, smoking status, alcohol use, moderate-to-vigorous physical activity, sleep duration, chronic disease history (diabetes, hypertension and cardiovascular disease) and food groups (dessert, fried food, fruit and vegetable) consumption (for SSB consumption only).

*P<0.05, **P<0.01, ***P<0.001.

Table S4 Associations of baseline sedentary behaviors and sugar-sweetened beverage consumption with obesity incidence in participants aged 6-20 years in Guangdong province in 2019-2021 and followed up till 2022

	**Participants with obesity, N (%)**	**Obesity, RR (95% CI)**		
		**Crude model**	**Model 1**	**Model 2**

Doing homework (x), hour/day				
0≤x<1	275 (2.53)	1.00	1.00	1.00
1≤x<2	508 (2.52)	1.00 (0.86, 1.15)	1.09 (0.94, 1.26)	1.08 (0.93, 1.25)
2≤x<3	328 (2.39)	0.94 (0.81, 1.11)	1.20 (1.02, 1.42) *	1.15 (0.97, 1.36)
x≥3	237 (2.20)	0.87 (0.73, 1.03)	1.21 (1.01, 1.45) *	1.15 (0.95, 1.38)
Attending tutorial class (x), hour/week				
0≤x<1	868 (2.28)	1.00	1.00	1.00
1≤x<2	212 (2.88)	1.27 (1.09, 1.47) **	1.07 (0.92, 1.25)	1.07 (0.92, 1.25)
2≤x<3	148 (2.86)	1.25 (1.06, 1.49) *	1.10 (0.93, 1.31)	1.04 (0.86, 1.25)
x≥3	156 (2.78)	1.22 (1.03, 1.45) *	1.15 (0.97, 1.36)	1.17 (0.98, 1.39)
Watching TV (x), hour/day				
0	239 (2.25)	1.00	1.00	1.00
0<x<1	536 (2.28)	1.01 (0.87, 1.18)	0.91 (0.78, 1.06)	0.93 (0.79, 1.09)
1≤x<2	360 (2.54)	1.13 (0.96, 1.33)	1.04 (0.89, 1.23)	0.97 (0.81, 1.17)
x≥2	289 (2.87)	1.28 (1.08, 1.51) **	1.19 (1.01, 1.41) *	1.05 (0.86, 1.28)
Computer use (x), hour/day				
0	644 (2.37)	1.00	1.00	1.00
0<x<1	425 (2.28)	0.97 (0.86, 1.09)	1.00 (0.89, 1.13)	0.99 (0.87, 1.13)
1≤x<2	202 (2.89)	1.22 (1.05, 1.43) *	1.25 (1.07, 1.47) **	1.12 (0.94, 1.33)
x≥2	153 (2.74)	1.16 (0.97, 1.38)	1.18 (0.99, 1.40)	1.04 (0.86, 1.27)
Mobile electronic device use (x), hour/day				
0	357 (2.62)	1.00	1.00	1.00
0<x<1	263 (2.14)	0.82 (0.70, 0.96) *	0.83 (0.71, 0.97) *	0.85 (0.72, 1.01)
1≤x<2	390 (2.59)	0.99 (0.86, 1.14)	1.08 (0.93, 1.25)	1.07 (0.91, 1.26)
x≥2	409 (2.36)	0.90 (0.78, 1.04)	1.29 (1.11, 1.50) **	1.23 (1.03, 1.46) *
Screen-related sedentary time, hour/day				
Per hour increment	1,411 (2.43)	1.02 (1.00, 1.03)	1.04 (1.02, 1.06) ***	1.04 (1.02, 1.05) ***
Academic study-related sedentary time, hour/day				
Per hour increment	1,314 (2.44)	1.03 (0.99, 1.06)	1.05 (1.01, 1.09) *	1.05 (1.01, 1.08) *
Total sedentary time, hour/day				
Per hour increment	1,303 (2.43)	1.01 (1.00, 1.03)	1.05 (1.03, 1.06) ***	1.04 (1.03, 1.06) ***
SSB consumption (x), time/day				
0	249 (2.40)	1.00	1.00	1.00
0<x<1	1,025 (2.39)	1.00 (0.87, 1.14)	1.06 (0.84, 1.35)	1.05 (0.82, 1.35)
x≥1	146 (2.82)	1.17 (0.96, 1.44)	1.65 (1.20, 2.27) **	1.59 (1.14, 2.22) **

N=number, RR=relative risk, CI=confidence interval, SSB=sugar-sweetened beverage.

Model 1: adjusted for sex, baseline age, ethnicity, urban/rural area, economic level, smoking status, alcohol use, moderate-to-vigorous physical activity, sleep duration, chronic disease history (diabetes, hypertension and cardiovascular disease) and food groups (dessert, fried food, fruit and vegetable) consumption (for SSB consumption only).

Model 2: mutually adjusted for total sedentary time and SSB consumption (for SSB consumption

and total or specific sedentary time). For specific sedentary behaviors, Model 2 was additionally mutually adjusted for specific sedentary behaviors.

*P<0.05, **P<0.01, ***P<0.001.

Table S5 Associations of baseline sedentary behaviors and sugar-sweetened beverage consumption with overweight incidence in participants aged 6-20 years in Guangdong province in 2019-2021 and followed up till 2022

	Participants with overweight, N (%)	Overweight, RR (95% CI)		
		**Crude model**	**Model 1**	**Model 2**
Doing homework (x), hour/day				
0≤x<1	574 (5.99)	1.00	1.00	1.00
1≤x<2	1,082 (6.13)	1.02 (0.93, 1.13)	1.09 (0.99, 1.20)	1.09 (0.98, 1.20)
2≤x<3	629 (5.26)	0.88 (0.79, 0.98) *	1.02 (0.92, 1.15)	1.02 (0.91, 1.14)
x≥3	509 (5.39)	0.90 (0.80, 1.01)	1.12 (0.99, 1.27)	1.11 (0.98, 1.26)
Attending tutorial classes (x), hour/week				
0≤x<1	1,805 (5.36)	1.00	1.00	1.00
1≤x<2	416 (6.53)	1.22 (1.10, 1.35) ***	1.10 (0.99, 1.22)	1.10 (0.99, 1.23)
2≤x<3	282 (6.33)	1.18 (1.05, 1.33) **	1.07 (0.95, 1.21)	1.09 (0.96, 1.23)
x≥3	323 (6.76)	1.26 (1.12, 1.41) ***	1.19 (1.06, 1.33) **	1.18 (1.04, 1.33) **
Watching TV (x), hour/day				
0	516 (5.53)	1.00	1.00	1.00
0<x<1	1,207 (5.81)	1.05 (0.95, 1.16)	1.00 (0.91, 1.11)	0.97 (0.87, 1.08)
1≤x<2	692 (5.61)	1.01 (0.91, 1.13)	0.96 (0.86, 1.07)	0.92 (0.81, 1.03)
x≥2	517 (5.94)	1.08 (0.96, 1.21)	1.03 (0.91, 1.16)	0.98 (0.85, 1.11)
Computer use (x), hour/day				
0	1,336 (5.58)	1.00	1.00	1.00
0<x<1	951 (5.81)	1.04 (0.96, 1.13)	1.04 (0.96, 1.13)	1.03 (0.94, 1.12)
1≤x<2	368 (6.08)	1.09 (0.97, 1.22)	1.08 (0.97, 1.21)	1.01 (0.90, 1.15)
x≥2	278 (5.81)	1.04 (0.92, 1.18)	1.01 (0.89, 1.15)	0.95 (0.82, 1.09)
Mobile electronic device use (x), hour/day				
0	685 (5.72)	1.00	1.00	1.00
0<x<1	644 (5.99)	1.05 (0.94, 1.16)	1.06 (0.95, 1.17)	1.04 (0.93, 1.16)
1≤x<2	759 (5.78)	1.01 (0.91, 1.12)	1.07 (0.96, 1.18)	1.06 (0.95, 1.18)
x≥2	836 (5.51)	0.96 (0.87, 1.06)	1.17 (1.05, 1.31) **	1.18 (1.05, 1.34) **
Screen-related sedentary time, hour/day				
Per hour increment	2,906 (5.71)	1.00 (0.98, 1.01)	1.01 (1.00, 1.02)	1.01 (0.99, 1.02)
Academic study-related sedentary time, hour/day				
Per hour increment	2,709 (5.74)	1.03 (1.00, 1.05) *	1.04 (1.01, 1.06) **	1.04 (1.01, 1.06) **
Total sedentary time, hour/day				
Per hour increment	2,683 (5.72)	1.00 (0.98, 1.01)	1.01 (1.00, 1.03) *	1.01 (1.00, 1.03)

SSB consumption (x), time/day				
0	507 (5.52)	1.00	1.00	1.00
0<x<1	2,152 (5.73)	1.04 (0.94, 1.14)	1.11 (0.94, 1.32)	1.13 (0.94, 1.34)
x≥1	272 (6.11)	1.11 (0.96, 1.28)	1.20 (0.96, 1.52)	1.10 (0.86, 1.41)

N=number, RR=relative risk, CI=confidence interval, SSB=sugar-sweetened beverage.

Model 1: adjusted for sex, baseline age, ethnicity, urban/rural area, economic level, smoking status, alcohol use, moderate-to-vigorous physical activity, sleep duration, chronic disease history (diabetes, hypertension and cardiovascular disease) and food groups (dessert, fried food, fruit and vegetable) consumption (for SSB consumption only).

Model 2: mutually adjusted for total sedentary time and SSB consumption (for SSB consumption and total or specific sedentary time). For specific sedentary behaviors, Model 2 was additionally mutually adjusted for specific sedentary behaviors.

*P<0.05, **P<0.01, ***P<0.001.

Table S6 Associations of baseline sedentary behaviors and sugar-sweetened beverage consumption with overweight/obesity incidence [a] in participants aged 6-20 years in Guangdong province in 2019-2021 and followed up till 2022

	**Participants with overweight/ obesity, N (%)**	**Overweight/obesity, RR (95% CI)**		
		**Crude model**	**Model 1**	**Model 2**
Doing homework (x), hour/day				
0≤x<1	606 (6.07)	1.00	1.00	1.00
1≤x<2	1,126 (6.11)	1.01 (0.92, 1.11)	1.12 (1.02, 1.24) *	1.12 (1.01, 1.23) *
2≤x<3	648 (5.20)	0.86 (0.77, 0.96) **	1.12 (1.00, 1.25) *	1.10 (0.99, 1.23)
x≥3	450 (4.60)	0.76 (0.67, 0.85) ***	1.11 (0.98, 1.25)	1.07 (0.94, 1.21)
Attending tutorial classes (x), hour/week				
0≤x<1	1,806 (5.17)	1.00	1.00	1.00
1≤x<2	453 (6.78)	1.31 (1.19, 1.45) ***	1.11 (1.00, 1.23) *	1.11 (1.00 1.23)
2≤x<3	285 (6.10)	1.18 (1.05, 1.33) **	1.03 (0.91, 1.16)	1.01 (0.89, 1.15)
x≥3	326 (6.52)	1.26 (1.13, 1.41) ***	1.20 (1.07, 1.34) **	1.18 (1.05, 1.33) **
Watching TV (x), hour/day				
0	496 (5.11)	1.00	1.00	1.00
0<x<1	1,215 (5.62)	1.10 (0.99, 1.22)	0.99 (0.89, 1.10)	0.95 (0.85, 1.06)
1≤x<2	732 (5.69)	1.11 (1.00, 1.24)	1.03 (0.92, 1.15)	0.96 (0.85, 1.08)
x≥2	529 (5.81)	1.14 (1.01, 1.28) *	1.08 (0.96, 1.21)	0.99 (0.87, 1.14)
Computer use (x), hour/day				
0	1,365 (5.48)	1.00	1.00	1.00
0<x<1	945 (5.55)	1.01 (0.93, 1.10)	1.05 (0.97, 1.14)	1.05 (0.96, 1.14)
1≤x<2	359 (5.68)	1.04 (0.93, 1.16)	1.10 (0.98, 1.23)	1.03 (0.91, 1.17)
x≥2	304 (6.05)	1.10 (0.98, 1.25)	1.14 (1.01, 1.29) *	1.03 (0.90, 1.18)
Mobile electronic device use (x), hour/day				
0	745 (5.96)	1.00	1.00	1.00
0<x<1	664 (5.94)	1.00 (0.90, 1.10)	1.03 (0.93, 1.14)	1.01 (0.91, 1.13)
1≤x<2	782 (5.71)	0.96 (0.87, 1.06)	1.07 (0.97, 1.18)	1.04 (0.93, 1.16)

x≥2	771 (4.89)	0.82 (0.74, 0.90) ***	1.21 (1.09, 1.35) ***	1.16 (1.03, 1.31) *
Screen-related sedentary time, hour/day				
Per hour increment	2,947 (5.56)	0.99 (0.98, 1.01)	1.02 (1.01, 1.03) ***	1.02 (1.01, 1.03) **
Academic study-related sedentary time, hour/day				
Per hour increment	2,750 (5.59)	1.01 (0.98, 1.03)	1.04 (1.01, 1.06) **	1.04 (1.01, 1.06) **
Total sedentary time, hour/day				
Per hour increment	2,725 (5.58)	0.99 (0.97, 1.00) *	1.03 (1.01, 1.04) ***	1.02 (1.01, 1.04) ***
SSB consumption (x), time/day				
0	555 (5.80)	1.00	1.00	1.00
0<x<1	2,138 (5.47)	0.94 (0.86, 1.03)	1.04 (0.88, 1.24)	1.04 (0.87, 1.24)
x≥1	276 (5.93)	1.02 (0.89, 1.18)	1.30 (1.03, 1.63) *	1.19 (0.93, 1.52)

N=number, RR=relative risk, CI=confidence interval, SSB=sugar-sweetened beverage.

[a] Overweight/obesity was defined by the age- and sex-specific BMI cut-off values from International Obesity Task Force (IOTF).

Model 1: adjusted for sex, baseline age, ethnicity, urban/rural area, economic level, smoking status, alcohol use, moderate-to-vigorous physical activity, sleep duration, chronic disease history (diabetes, hypertension and cardiovascular disease) and food groups (dessert, fried food, fruit and vegetable) consumption (for SSB consumption only).

Model 2: mutually adjusted for total sedentary time and SSB consumption (for SSB consumption and total or specific sedentary time). For specific sedentary behaviors, Model 2 was additionally mutually adjusted for specific sedentary behaviors.

*P<0.05, **P<0.01, ***P<0.001.

Table S7 Comparison of baseline characteristics between participants included and not included in the main analysis of the association between total sedentary time and overweight/obesity incidence

	Participants included in the analysis	Participants not included in the analysis	Cohen's effect size [a]
Number of participants, N (%)	47,148 (74.67)	15,991 (25.33)	-
Age, years, mean (SD)	12.39 (2.86)	12.02 (2.72)	0.13
Sex, %			0.10
Men	49.20	60.68	
Women	50.80	39.32	
Ethnicity, %			0.01
Han	98.17	97.95	
Others	1.83	2.05	
Area, %			0.01
Urban	55.04	56.60	
Rural	44.96	43.40	
Economic level, %			0.02

Low	36.96	35.93	
Middle	31.60	30.10	
High	31.44	33.97	
Smoking status, %			0.02
Never	95.34	94.51	
Former	3.98	4.35	
Current	0.68	1.14	
Alcohol use, %			0.02
No	81.75	79.79	
Yes	18.25	20.21	
MVPA frequency, day/week			0.02
0-1	29.35	30.17	
2-3	34.31	33.01	
4-5	18.20	17.63	
6-7	18.15	19.18	
Diabetes, %			0.01
No	99.97	99.94	
Yes	0.03	0.06	
Hypertension, %			0.01
No	99.98	99.95	
Yes	0.02	0.05	
Cardiovascular disease, %			0.01
No	99.90	99.92	
Yes	0.10	0.08	
Sleep duration, hour/day, mean (SD)	8.27 (1.83)	8.32 (2.07)	0.02

N=number, SD=standard deviation, MVPA=moderate-to-vigorous physical activity.

[a] Cohen's d value was used for continuous variables, while Cohen's w value was used for categorical variables. Cohen classified effect sizes as small (d=0.2, or w=0.1), medium (d=0.5, or w=0.3), and large (d≥0.8 or w≥0.5).

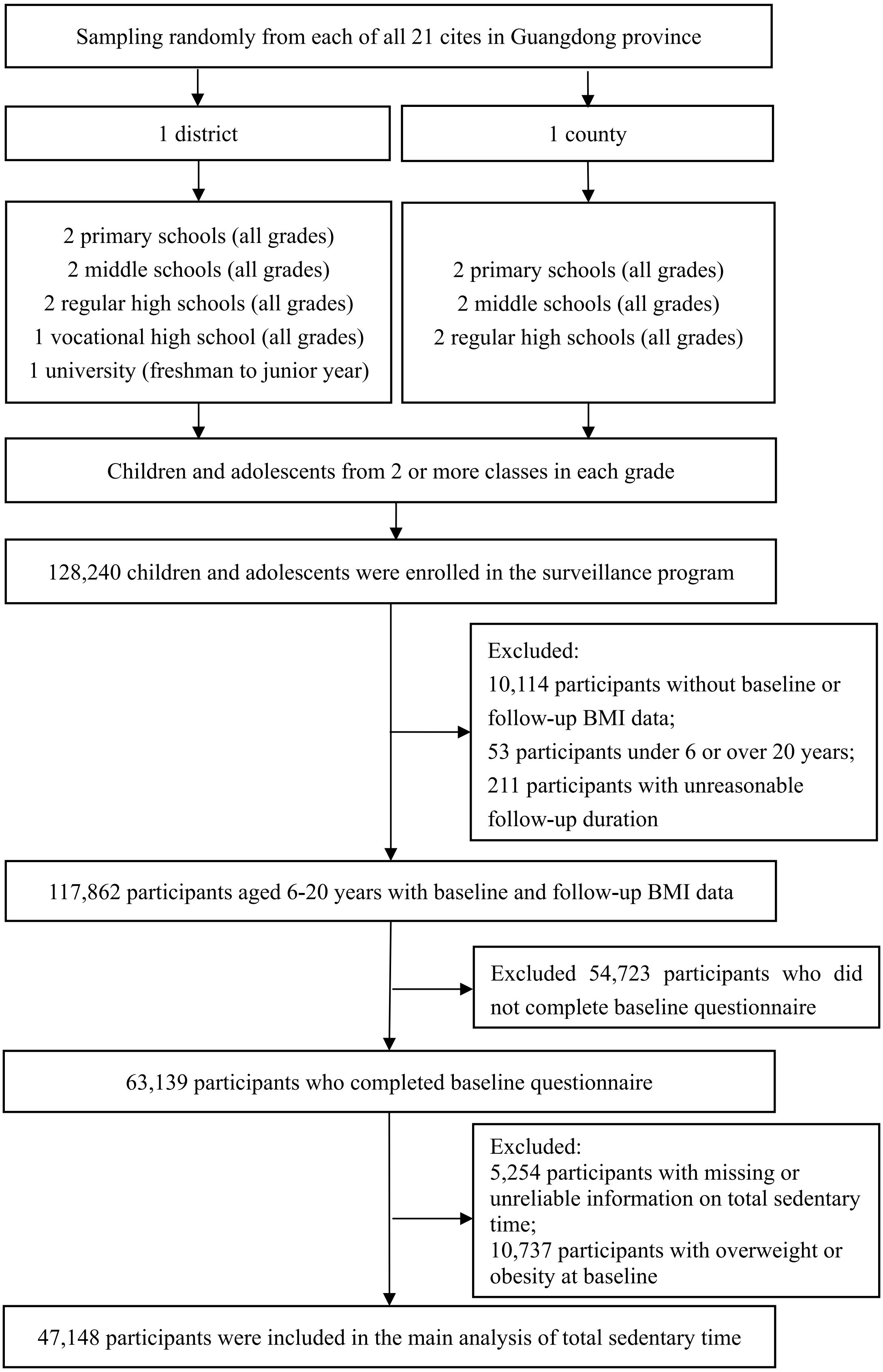

Figure S1 Flow chart of surveillance design and participants selection in the main analysis
BMI=body mass index.

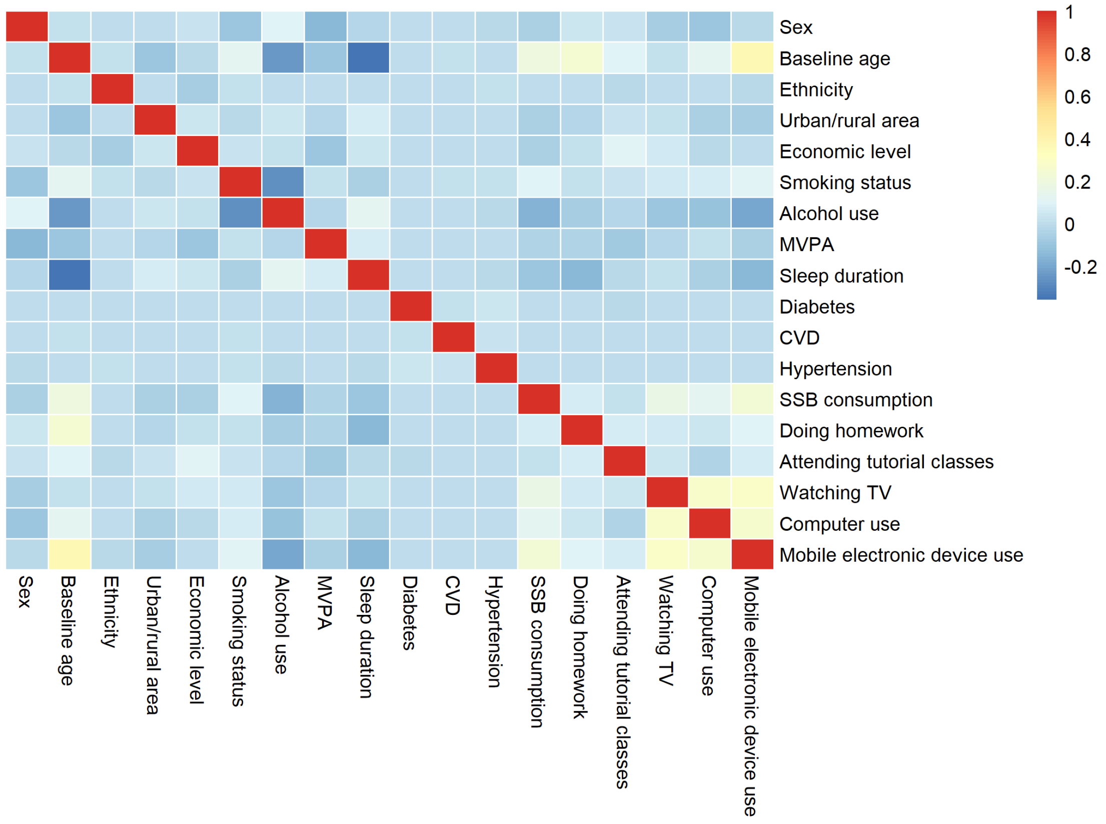

Figure S2 Heatmap of correlation coefficients among variables included in the mutually adjusted model for specific sedentary behaviors

MVPA=moderate-to-vigorous physical activity, CVD=cardiovascular disease, SSB=sugar-sweetened beverage.

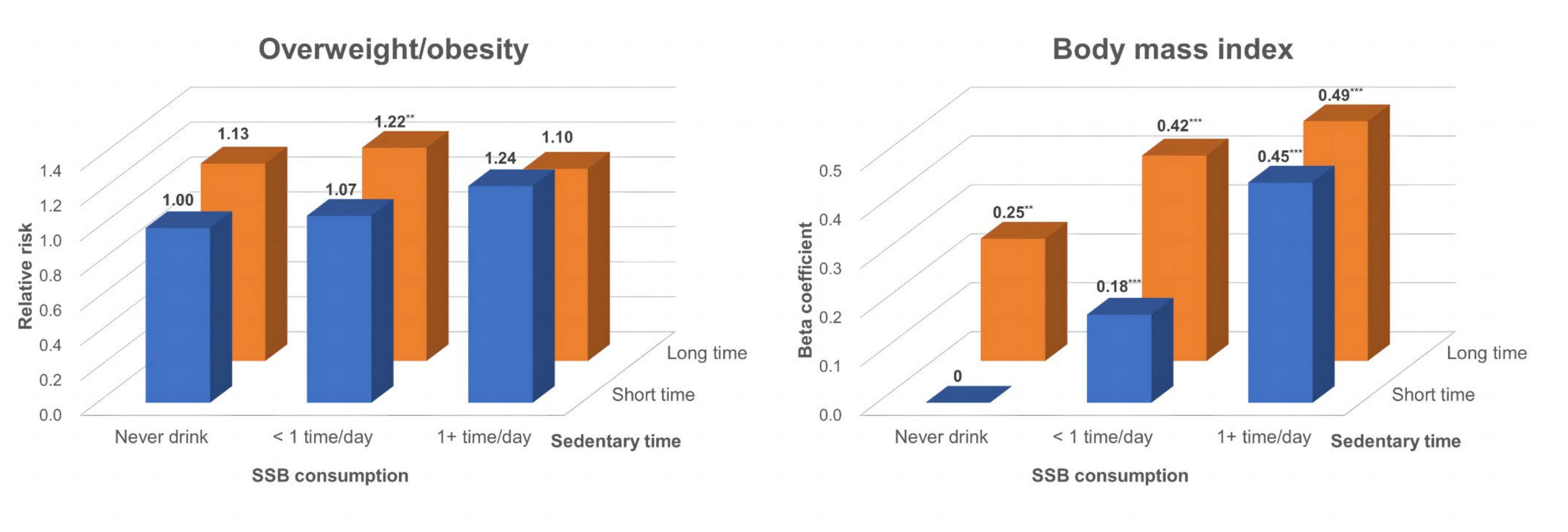

Figure S3 Adjusted relative risks/βs [a] for overweight/obesity incidence and followed-up body mass index (BMI) by groups of total sedentary time and sugar-sweetened beverage consumption in participants aged 6-20 years in Guangdong province in 2019-2021 and followed up till 2022

SSB=sugar-sweetened beverage.

[a] Relative risks/βs were adjusted for sex, baseline age, ethnicity, urban/rural area, economic level, smoking status, alcohol use, moderate-to-vigorous physical activity, sleep duration and chronic disease history (diabetes, hypertension and cardiovascular disease).

$^{*}P<0.05$, $^{**}P<0.01$, $^{***}P<0.001$.
